# Thermodynamic Studies of Interactions between Sertraline Hydrochloride and Randomly Methylated β-Cyclodextrin Molecules Supported by Circular Dichroism Spectroscopy and Molecular Docking Results

**DOI:** 10.3390/ijms222212357

**Published:** 2021-11-16

**Authors:** Sylwia Belica-Pacha, Mateusz Daśko, Vyacheslav Buko, Ilya Zavodnik, Katarzyna Miłowska, Maria Bryszewska

**Affiliations:** 1 Unit of Biophysical Chemistry, Department of Physical Chemistry, Faculty of Chemistry, University of Lodz, Pomorska 165, 90-236 Lodz, Poland; 2 Department of Inorganic Chemistry, Faculty of Chemistry, Gdansk University of Technology, Narutowicza 11/12, 80-233 Gdansk, Poland; mateusz.dasko@pg.edu.pl; 3 Division of Biochemical Pharmacology, Institute of Biochemistry of Biologically Active Compounds, National Academy of Sciences, BLK-50, 230030 Grodno, Belarus; vu.buko@tut.by (V.B.); zavodnik_il@mail.ru (I.Z.); 4 Department of Biotechnology, School of Medical Sciences, Krakowska 9, 15-875 Bialystok, Poland; 5 Department of Biochemistry, Yanka Kupala Grodno State University, BLK-50, 230030 Grodno, Belarus; 6 Department of General Biophysics, Faculty of Biology and Environmental Protection, University of Lodz, Pomorska 141/143, 90-236 Lodz, Poland; katarzyna.milowska@biol.uni.lodz.pl (K.M.); maria.bryszewska@biol.uni.lodz.pl (M.B.)

**Keywords:** sertraline hydrochloride, β-cyclodextrin, methylated β-cyclodextrin, isothermal titration calorimetry, circular dichroism, molecular docking

## Abstract

The interaction between sertraline hydrochloride (SRT) and randomly methylated β-cyclodextrin (RMβCD) molecules have been investigated at 298.15 K under atmospheric pressure. The method used—Isothermal Titration Calorimetry (ITC) enabled to determine values of the thermodynamic functions like the enthalpy (ΔH), the entropy (ΔS) and the Gibbs free energy (ΔG) of binding for the examined system. Moreover, the stoichiometry coefficient of binding (n) and binding/association constant (K) value have been calculated from the experimental results. The obtained outcome was compared with the data from the literature for other non-ionic βCD derivatives interacting with SRT and the enthalpy-entropy compensation were observed and interpreted. Furthermore, the connection of RMβCD with SRT was characterized by circular dichroism spectroscopy (CD) and complexes of βCD derivatives with SRT were characterized through the computational studies with the use of molecular docking (MD).

## 1. Introduction

Cyclodextrins are water-soluble cyclic oligo-saccharides with a truncated cone-structure possessed a hydrophobic cavity and hydrophilic outer surface border [1]. Recently, the guest-host cyclodextrin complex formations were examined for many and different ligands like for example: remdesivir [2], water-soluble betulin derivatives [3], thiabendazole [4], ethinyloestradiol [5], bis(1,10-phenanthroline) silver (I) salicylate [6], oncocalyxone A [7], or β-cyclodextrin-enhanced Eu^3+^ luminescence aggregates with bright red fluorescence of Eu^3+^ convenient for environmental detection system [8] and many others [9,10,11,12]. Reactions involving cyclodextrins are still important to the separation [13,14] and food industry [15,16,17] or other kinds of industries [18,19], but mostly to drug delivery systems for various applications in the field of medical biomaterials for example in the technology of polymers to get artificial abdominal wall prosthesis textile with improved biological and antibiotic delivery properties [20]. The basis for advanced tests of the cyclodextrins application are still information about inclusion complexation by natural and modified cyclodextrins, as well as the factors involved in controlling the supramolecular interactions [21]. Lately, the interactions between antidepressant drugs and cyclodextrins were examined [22,23,24,25,26], mostly in order to gain the better water solubility of the drug [27,28,29], or to decrease its toxicity [30,31] or even for taste-masking [32]. One of the antidepressants, sertraline hydrochloride, belonging to the class of selective serotonin reuptake inhibitors (SSRIs) [33,34] and also tested as potential anti-*Helicobacter pylori* compound [35] has already been tested in the presence of β-cyclodextrin and the derivatives [36,37,38,39,40] inter alia in our laboratory [31,41,42].

**Figure 1:**
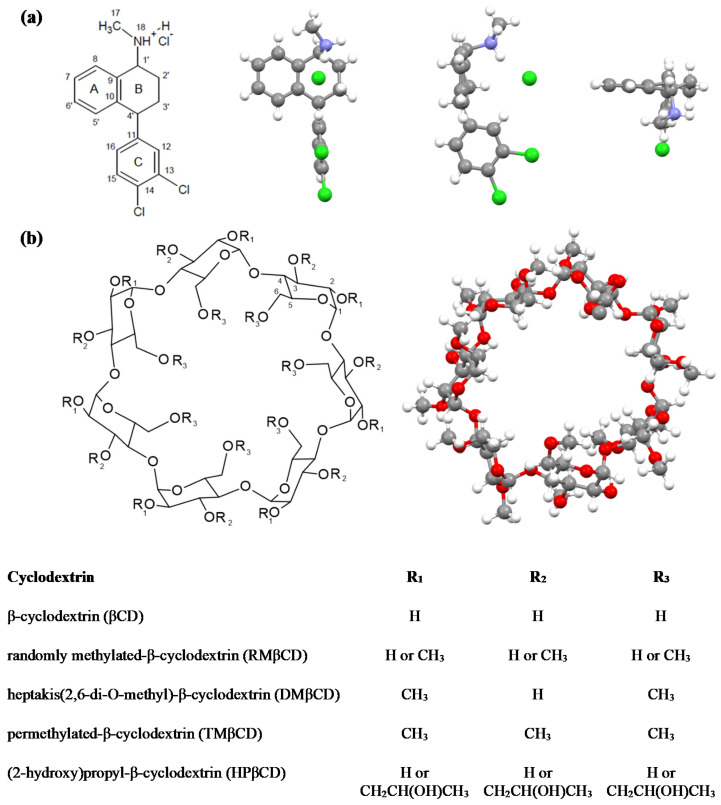
(**a**) Sertraline hydrochloride structural formula (on the **left**) and the models made based on crystal structure of sertraline hydrochloride (on the **right**) with refcode CAVVUQ [46] from the Cambridge Structural Database (CSD) [47] and (**b**) a general structural formula of some β-cyclodextrins (on the **left**) and crystal structure of RMβCD (on the **right**) with refcode JOSWOD [48] from the CSD [47].

The aim of presented work was to obtain information about interactions of sertraline hydrochloride (SRT) (Figure 1a) with randomly methylated β-cyclodextrin (RMβCD) (Figure 1b) in water solutions at 298.15 K under atmospheric pressure. The binding process of guest molecule with cyclodextrin was examined with the use of an isothermal titration calorimetry (ITC) [43,44,45] and molecular docking studies (MD) [10,43,45]. The outcome was compared with the set of data for other complexes of SRT with non-ionic β-cyclodextrin derivatives (Figure 1b) received from the literature [31,41,42]. For the thermodynamic results the enthalpy-entropy compensation has been observed. In the near future, we are going to check, if similar compensation is observed for interactions of SRT with ionic β-cyclodextrin derivatives. We have also decided to use the circular dichroism spectroscopy (CD) experimental method to thorough examinations of the SRT:RMβCD complex formation and to confirm the stoichiometry coefficient (n) and binding/association constant (K) value calculated from ITC experimental data.

## 2. Results and Discussion

### 2.1. Isothermal Titration Calorimetry (ITC)

**Figure 2:**
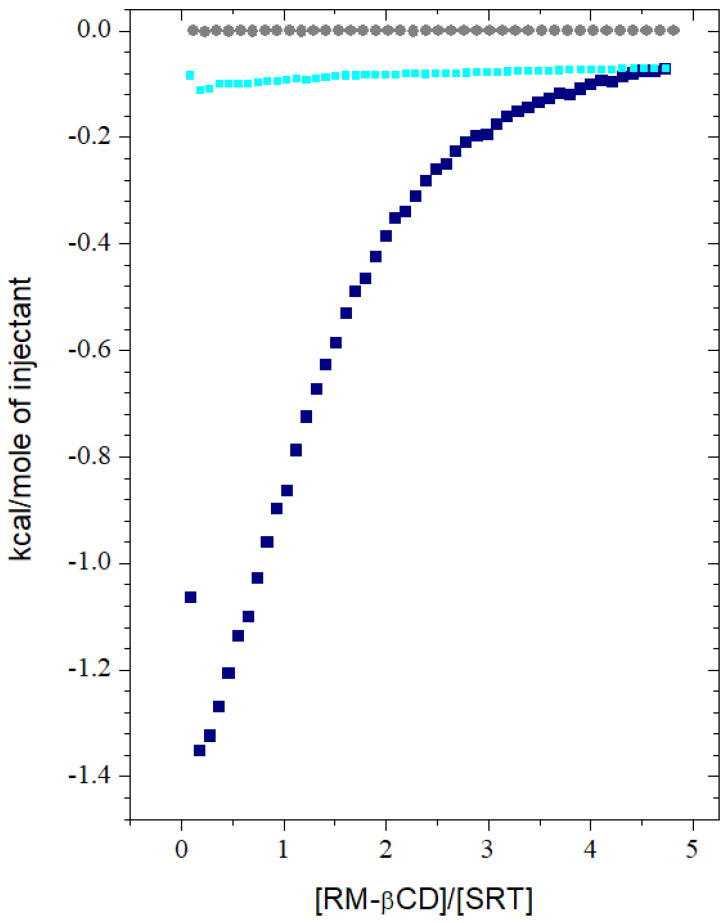
The integrated thermal effects corresponding to the binding interaction during titration of a 0.45 mM sertraline hydrochloride solution (in a cell) with a 15 mM solution of randomly methylated-β-cyclodextrin (in a syringe) (■ navy blue) for aqueous solutions with pH ≈ 6.8 at 298.15 K under atmospheric pressure *p* = 101,800 Pa together with the effects of SRT (●gray) and RMβCD (■ cyan) dilution by pure water.

An isothermal titration calorimetry method has been chosen in order to get a full package of thermodynamic functions values. An enthalpic (ΔH) and an entropic (TΔS) contribution into the Gibbs free energy value (ΔG), together with stoichiometry coefficient (n) and binding constant (K) could be determined by recording a heat change during direct chemical interaction between the sertraline hydrochloride and the randomly methylated-β-cyclodextrin molecules in water solution. An example of a typical experimental result has been placed in Figure 2.

The “One-set of independent sites model” [49,50] was selected among available in the ORIGIN 7 program options [51,52] to get the thermodynamical parameters (Table 1). This selection was justified inter alia in the least errors of the fitted parameters [53] in comparison to results obtained from the other models like e.g., “Two-sets of sites” [53,54]. Moreover, the first point (Figure 2) from first injection during the fitting sessions was discarded considering the circumstances described previously [26].

**Figure 3:**
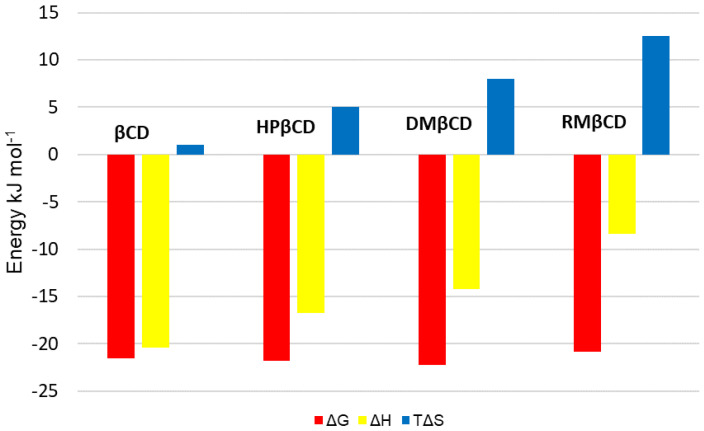
Binding free Gibbs energy (ΔG), enthalpy (ΔH), and entropy factor (TΔS) of inclusion complex formation between SRT and: βCD [41], DMβCD [41], HPβCD [42], RMβCD (this work).

The complex formation between SRT and RMβCD molecules represents a rather strong interaction since the association constant value (K) is greater than 1000 M^−1^ [49], but in comparison with analogous interactions obtained for SRT and other βCDs (Table 1), the K value for RMβCD is the smallest. Furthermore, the energetic effects of interaction between RMβCD and SRT molecules are exothermic like for the rest compared SRT:βCDs systems, for which the enthalpy ΔH values are all less than zero (Table 1, Figure 3). It is worth to mention, that only for RMβCD and SRT molecules the enthalpic effects of binding have been dominated by the entropic effects (|ΔH| < |TΔS|) occurring during the drug-cyclodextrin complex formation (Figure 3).

Thus, the entropy factor is quite significant for SRT:RMβCD complex formation and seems to be connected with the release of water molecules that were originally installed in the cavity of the cyclodextrin and the desolvation of peripheral hydroxyl or substituted hydroxyl groups as well as the guest molecules [55]. Once again, the SRT:RMβCD complexation is driven both by the enthalpy and the entropy factors, but the entropic term controls the complex’s ultimate stability. 

**Figure 4:**
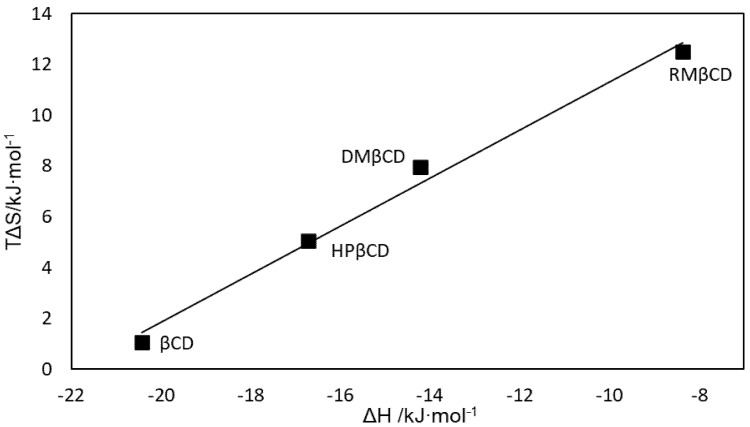
The enthalpy-entropy compensation plot for inclusion complexes of sertraline hydrochloride molecules with chosen β-cyclodextrin molecules: βCD [41], DMβCD [41], HPβCD [42], RMβCD (this work). The determined coefficients of the linear equation TΔS = α∙ΔH + TΔS_0_ [21] with R^2^ = 0.99 are: α = (0.946 ± 0.068) kJ∙mol^−1^ and TΔS_0_ = (20.8 ± 1.1) kJ∙mol^−1^.

Moreover, the absolute value of Gibbs free energy of binding (Table 1) for SRT:RMβCD system turned out to be the smallest one. In addition, the complexation processes for all interactions of SRT with chosen β-cyclodextrins are spontaneous—the values of the Gibbs free energies of binding are all less than zero (Table 1, Figure 3). The maximum Gibbs free energy value change (ΔΔG) equals 1.33 kJ mol^−1^ and can be observed between ΔG values for SRT:DMβCD and SRT:TMβCD complexes. These rather small differences in ΔG values (less than or close to 1 kJ mol^−1^, Table 1, Figure 3) observed for interactions of SRT with chosen non-ionic β-cyclodextrins are qualitatively the source of the ΔH-ΔS compensation effect. Leffler stated [56], that in a series of related processes involving moderate changes in structure or solvent, the enthalpies and entropies vary, but usually not independently. Moreover, in the cited work [56] the author observed that the correlation of enthalpy with the entropy factor may approach almost linear relationship for the series of interactions studied. In the work presented, such a regularity has been observed for the non-ionic series of β-cyclodextrin derivatives interacted with sertraline hydrochloride molecules, which can be seen on Figure 4. The observed empirical dependence of the compensating enthalpy-entropy effects can be described by Equation (1) [21], where α is a slope of the T∆S-vs-∆H plot and T∆S_0_ is an intercept of the plot (Figure 4)

T∆S = α∆H + T∆S_0_ (1)

The slope (α) of the graph indicates to what extent the enthalpy factor caused by changes in the structure of the host, guest and/or solvent is eliminated by the accompanying entropy effects [21]. Mostly, the literature data for the α slope of cyclodextrins extend over the range from 0.79 to 0.88 [21] or closer to unity, when modified cyclodextrins possess greater ring flexibility and/or flexible side chains [57], but there are also known examples of incomplete enthalpy-entropy compensation effect, when the slope is even 0.53 [58]. In the presented study, the obtained value is α = (0.946 ± 0.068) kJ∙mol^−1^ (Figure 4).

It is believed that the large slope, as shown in Figure 4, is caused by the rearrangement of the peripheral network of hydrogen bonds with the accompanying conformational changes in the skeleton [59]. It means, that only a fraction of the enthalpy effect, equal to the difference (1 − α), contributes to increasing the stability of the complex. In the studied case, it is ca. 5% of the enthalpic factor induced by system modifications, which have a share of the ΔG change (ΔΔG) [55]. Such a value is expected due to the relatively high rigidity of the cyclic cyclodextrin structure [21]. On the other hand, the intercept T∆S_0_ represents the intrinsic stability of the complex obtained for ΔH = 0. For the relationship presented on Figure 4, the T∆S_0_ is positive and indicates that the complex will be stabilized even in the absence of the favorable enthalpy effects [21,60]. The literature intercept values (T∆S_0_) obtained for cyclodextrins are mostly within the range 8–17 kJ mol^−1^ [21], but for those with flexible hydrophilic substituents, the value is 21 kJ mol^−1^ [21] and that calculated from our experimental data, 20.8 ± 1.1 kJ∙mol^−1^, is almost the same or the same in the range of experimental error. Consequently, the resulting slope and intersection point obtained for selected non-ionic βCD interacting with SRT can be attributed to greater conformational changes and increased desolvation compared to the interaction of native β-cyclodextrin with the SRT molecule [55].

Once again, the binding constant of SRT inclusion inside the RMβCD cavity is lower than for other βCDs (Table 1) even for DMβCD, which molecule has the same methyl groups as substituents in the βCD molecule, but for the RMβCD they are placed randomly. Probably, this uneven distribution of methyl groups around the wider and narrower cyclodextrin edges has a certain destabilizing effect during the guest-host complex formation. Presumably, it is connected with the less enthalpy gain in the free Gibbs energy. The van der Waals interactions between the SRT and RMβCD molecules are most likely limited by the less suitable fitting of the SRT molecule inside the RMβCD cavity, since van der Waals forces are critically dependent on the distance of separation [21]. Moreover, the possible hydrogen bonds between the SRT and the hydroxyl groups of RMβCD cannot form, because the random substitution has blocked them. However, the change in the constant K value due to these perturbations is generally much smaller than might be expected from the change in the enthalpy effects themselves, since the effects have been largely compensated by a significant proportion of the changing entropy effects. 

The stoichiometry coefficient n (Table 1) of the complex formation between randomly methylated-β-cyclodextrin and the sertraline hydrochloride molecules in water solution indicates the ratio for RMβCD:SRT above 1, even if some humidity degree of the macromolecule was taken into account in the concentration calculations [61,62]. The coefficient values (n) for the remaining complexes are similar to the stoichiometry of RMβCD:SRT adduct and slightly higher for the DMβCD:SRT complex. These results suggest that 1:1 connection co-existing in the water solution with adducts of more than one molecule of βCD (or its derivative) with one SRT molecule. In order to confirm the suggestion, the circular dichroism examinations and molecular docking simulations of βCDs interacting with SRT molecules were conducted and the results are presented below.

### 2.2. Circular Dichroism Spectroscopy (CD)

**Figure 5:**
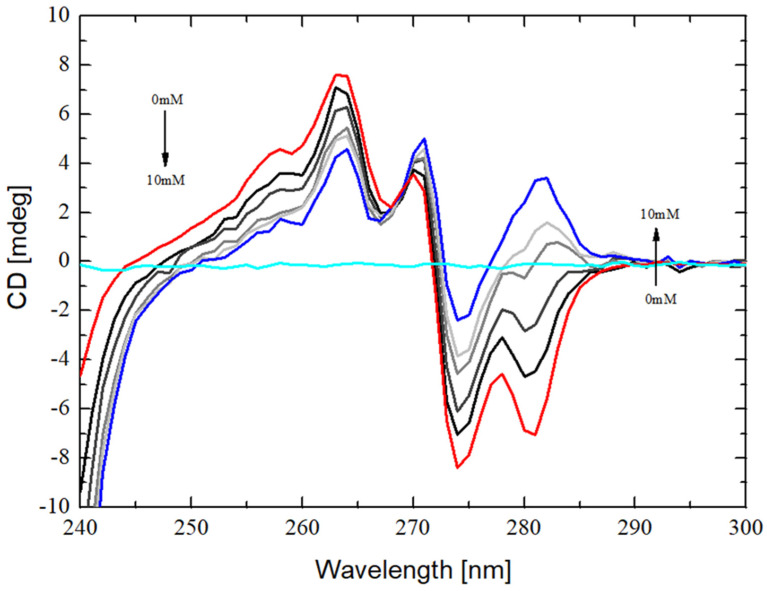
The circular dichroism spectrum of 0.3 mM sertraline hydrochloride aqueous solution (red line) together with spectrum of the 0.3 mM randomly methylated-β-cyclodextrin aqueous solution (cyan line) and the spectra of drug-cyclodextrin mixtures (from black with ratio of 1:0.5 thru the ratio 1:1, 1:2 to light gray with ratio of 1:3 for SRT:RMβCD) with constant concentration of SRT and growing content of RMβCD to the maximum concentration of 10 mM (blue line with ratio of 1:33).

The water solution of sertraline hydrochloride presents an optical activity as a spectrum with characteristic circular dichroism bands (Figure 5, red line)—they are: a shoulder at 258 nm, two positive peaks (positive Cotton effects) at 263 nm and 270 nm, two negative peaks (negative Cotton effects) at 274 nm and 280 nm. Most of the peaks decrease in the intensity in the presence of randomly methylated β-cyclodextrin (Figure 5, from the black to the light gray line), which itself does not show any significant circular dichroism bands (Figure 5, cyan line). Only the peak intensity at the 270 nm wavelength increases and shifts towards longer wavelengths values with increasing concentration of the cyclodextrin.

The changes in the CD spectra of SRT + RMβCD mixtures in comparison to pure SRT spectrum indicate that the sertraline hydrochloride molecules interact with the cyclodextrin [63]. These effects may be induced by the entrance of the guest molecule into the optically active RMβCD cavity [64]. The parallel orientation of the electric transition dipole moment of the drug molecule towards the molecular z-axis of the cyclodextrin molecule induced the positive circular dichroism [65] like the observed growing band for SRT:RMβCD mixture at 270 nm (Figure 5). 

Moreover, negative peak at 280 nm becomes flatter and transform into a positive shoulder and a new positive peak at 281 nm in the presence of RMβCD at the maximum concentration (Figure 5). The positive peak at 281 nm becomes noticeable already for the 2:1 ratio of RMβCD:SRT, which might suggest that the A (together with B) and the C ring of SRT molecule (Figure 1a) enter separately into the cavities of two different RMβCD molecules [63]. In other words, the registered transformation of the negative band at 281 nm into a positive one, along with an increase in the excess of the cyclodextrin in relation to the drug, may indicate the formation of complexes with a stoichiometry greater than 1:1.

**Figure 6:**
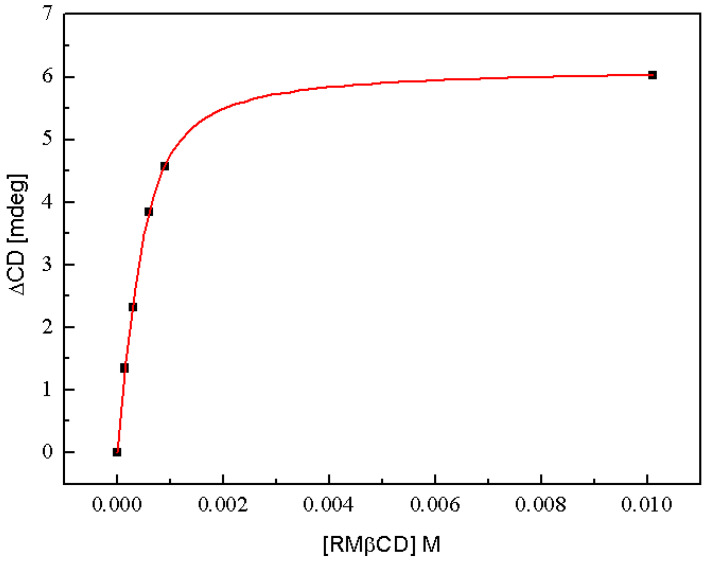
The change in circular dichroism intensity (ΔCD) of SRT at 274 nm as a function of RMβCD concentration. The red solid line represents the best fit of Equation (2) to the experimental results presented as the black points and the parameters from fitting calculations are placed in Table 2.

To check the stoichiometry of RMβCD:SRT complexes (n) as well as to determine the value of the association constant (*K*) based on the circular dichroism results, one can use the equation proposed originally for NMR results by Fielding [66] given below:

(2) $$\Delta CD=\frac{\Delta CD_{max}}{2}\left[\left(1+\frac{\left[RM\beta CD\right]}{n\left[SRT\right]}+\frac{1}{Kn\left[SRT\right]}\right)-{\left({\left(1+\frac{\left[RM\beta CD\right]}{n\left[SRT\right]}+\frac{1}{Kn\left[SRT\right]}\right)}^{2}-\frac{4\left[RM\beta CD\right]}{n\left[SRT\right]}\right)}^{0.5}\right]$$

where: *ΔCD* is the change in circular dichroism intensity, [*RMβCD*] and [*SRT*] are the appropriate molar concentrations and *ΔCD_max_*, n and *K* are the parameters obtained from non-linear regression analysis (Figure 6). From the results calculated based on Equation (2) and the circular dichroism intensities bands (Figure 5), the best fit has been selected, which was at 274 nm, and placed in Table 2.

The stoichiometry coefficient of RMβCD:SRT complex formation determined on the basis of the CD data (*n* = 1.57 ± 0.11) reaffirms the stoichiometry coefficient estimated by the ITC method (*n* = 1.26 ± 0.05). The convergence of these data once more suggests that in the resulting complex there is more than one molecule of the cyclodextrin per one drug molecule. Furthermore, the obtained value of the constant *K* (Table 2) differs by only 800 units in relation to that determined from the ITC research (Table 1), which can be considered as a slight difference bearing in mind the accuracy of both experimental methods. 

### 2.3. Molecular Docking (MD)

The molecular docking studies have been carried out for more in-depth analysis of the interactions between the test sertraline hydrochloride and the randomly methylated β-cyclodextrin as well as the other selected non-ionic cyclodextrins. The simulations were carried out on the basis of the available crystal structures, hence no molecular docking studies for (2-hydroxy)propyl-β-cyclodextrin were carried out, because there is no crystallographic data for this cyclodextrin in the Cambridge Structural Database (CSD) [47]. 

**Figure 7:**
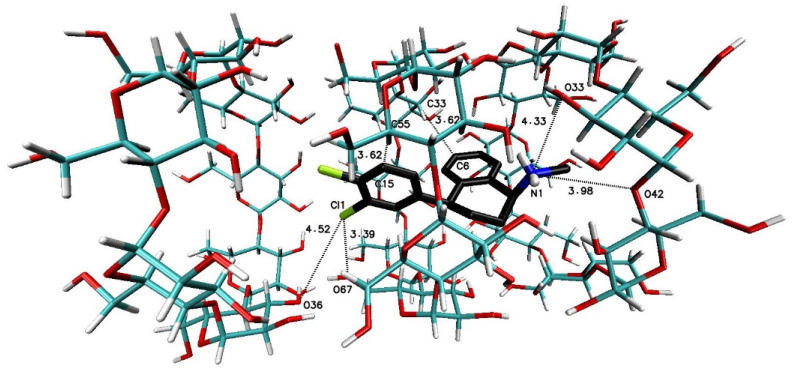
The geometry of the SRT-βCD complex with stoichiometry 1:3 (I-II-III or “head-to-head-to-tail” as in [69,70]) obtained by the Molecular Docking (MD) simulations with the use of βCD crystal structure with refcode 648855 [67] from The Cambridge Structural Database (CSD) [47].

**Figure 8:**
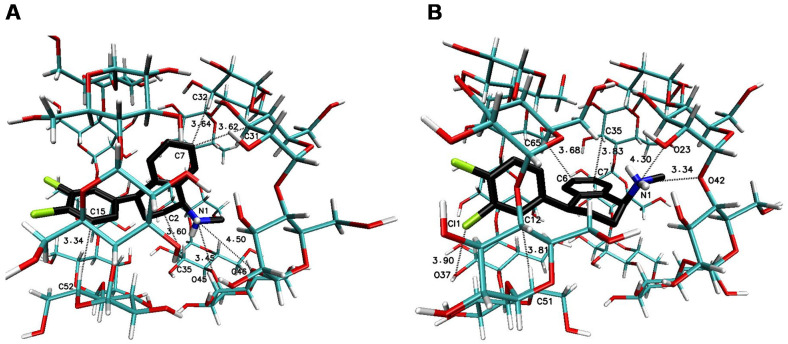
The geometries of the SRT-βCD complex with stoichiometry 1:2 (I-II for (**A**) or “head-to-head” [69,70] and II-III or “head-to-tail” for (**B**) obtained by MD simulations with the use of βCD crystal structure with refcode 648855 [67] from the CSD database [47].

**Figure 9:**
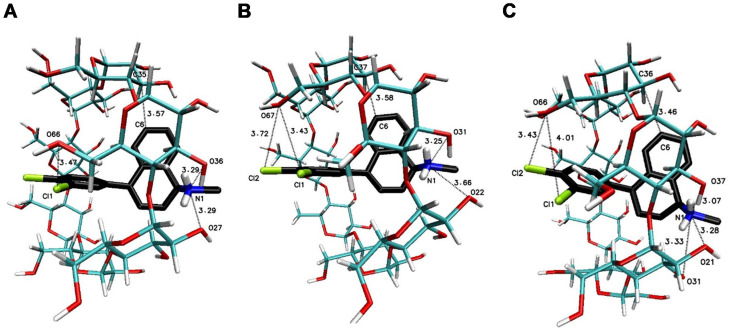
The geometries of the SRT-βCD complex with stoichiometry 1:1-I for (**A**), II for (**B**) and III for (**C**)-obtained by MD simulations with the use of βCD crystal structure with refcode 648855 [67] from the CSD database [47].

**Figure 10:**
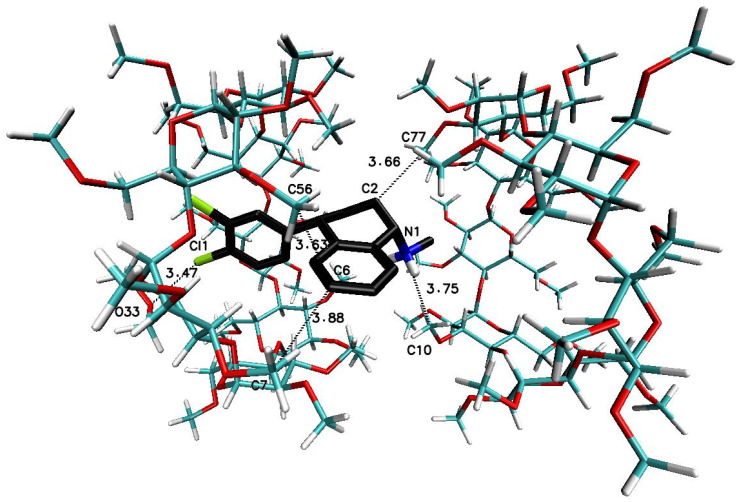
The geometries of the SRT-TMβCD complex with stoichiometry 1:2 (I-II or “head-to-head” [69,70]) obtained by MD simulations with the use of TMβCD crystal structure with refcode ALIGAE [68] from the CSD database [47].

**Figure 11:**
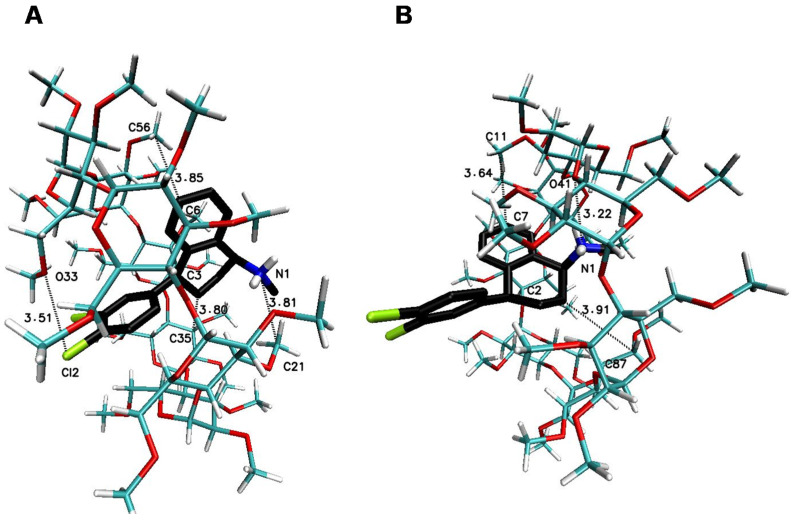
The geometry of the SRT-TMβCD complex with stoichiometry 1:1 (I for (**A**) and II for (**B**) structure) obtained by MD simulations with the use of TMβCD crystal structure with refcode ALIGAE [68] from the CSD database [47].

**Figure 12:**
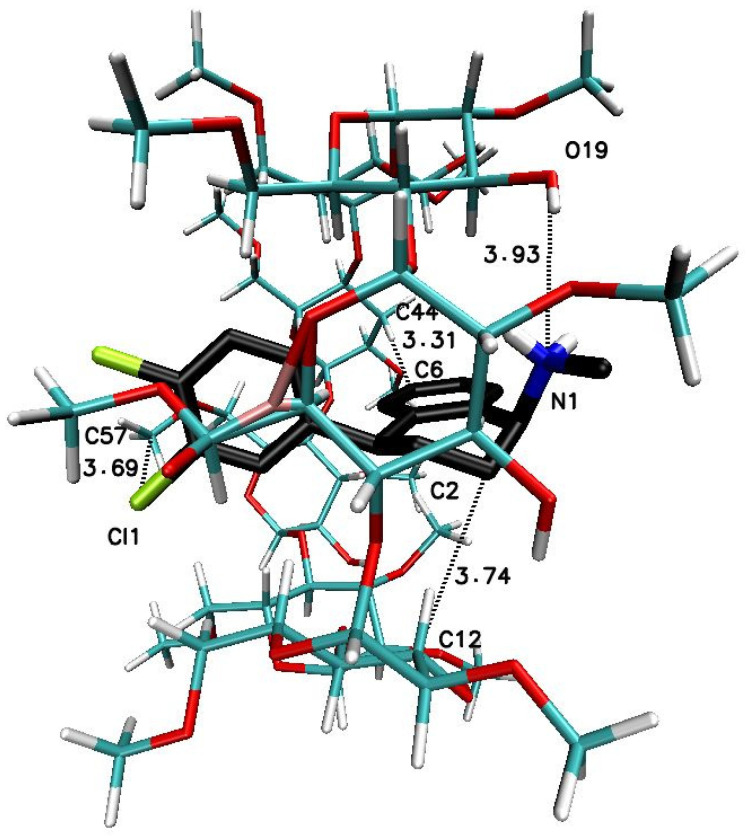
The geometry of the SRT-RMβCD complex with stoichiometry 1:1 obtained by MD simulations with the use of RMβCD crystal structure with refcode JOSWOD [48] from the CSD database [47].

As well, the results for sertraline hydrochloride docking inside the heptakis(2,6-di-O-methyl)-β-cyclodextrin molecule will be carried out and published separately in the future work. It should also be emphasized that crystal structures for β-cyclodextrin and permethylated-β-cyclodextrin have more than one cyclodextrin molecule in an independent cell and there are three for βCD [67] and two for TMβCD [68]. Such a three-molecule structure of βCD (or two-molecule of TMβCD) can be used to check what the free energy of binding has the resulting complex with 1:3 SRT:βCD (or 1:2 for SRT:TMβCD) stoichiometry and whether this energy is the optimal value for possible complexes [69]. Unfortunately, it was not possible to check the energy of RMβCD with SRT complexes with a stoichiometry greater than 1:1, because the available crystal structure of this cyclodextrin has only one molecule in its independent cell [48]. To obtain results comparable to those for SRT docking in RMβCD, the remaining 1:1 type complex was obtained by stepwise removal of successive βCD (or TMβCD) molecules from the structure and re-optimization of the resulting complex was performed [26] and the results of all molecular docking studies are presented in Table 3 and Figure 7, Figure 8, Figure 9, Figure 10, Figure 11 and Figure 12.

The significant difference in the magnitude of the binding energies can be noticed for the 1:3 SRT:βCD complex in comparison to other energy of binding values (Table 3). On the Figure 7 have been placed example of the SRT-βCD complex geometry with stoichiometry 1:3 obtained by MD simulations with the use of βCD crystal structure and as it seen, the guest molecule of sertraline freely penetrates at least two host molecules with some probability to reach the third one. This is noticeable as well as in the binding energy absolute value reduction (with difference of 2 kJ∙mol^−1^) of the four-molecule complex (SRT + 3βCD with the position of βCD molecules named I-II-III or “head-to-head-to-tail” as in [69,70]) after removal one of the βCD molecules and re-optimization of the binding energy for SRT + 2βCD aggregate (Table 3). Adducts of SRT with two βCD host molecules represent still rather stable systems with some differences in the binding energies for different host molecules configurations (Figure 8, Table 3 for A represented I-II or “head-to-head” and B represented II-III or “head-to-tail” structure) and this is also the case of two TMβCD molecules interacting with SRT molecule (Figure 10, Table 3). For all other 1:1 SRT:CD structures, regardless of the type of the cyclodextrin, the binding free energy values are the lowest and differ from each other by a maximum of 0.8 kcal∙mol^−1^ (3 kJ∙mol^−1^) (Table 3 and Figure 9 for structures A–C and Figure 11 for A and B and Figure 12). 

When interpreting molecular docking studies, it should be borne in mind that they are very approximate and serve as preliminary research. In order to obtain more reliable results in this respect, they should be repeated using semi-empirical methods, such as i.a. GFN2-xTB [71] and clarified with DFT research [25,71]. The mentioned examinations are in our plans for the next part of our work focusing more on quantum-chemical calculations, which we have already conducted before with very good results in the mianserin hydrochloride + β-cyclodextrin system [25].

## 3. Materials and Methods

### 3.1. Materials

Sertraline hydrochloride (1S,4S)-4-(3,4-dichlorophenyl)-1,2,3,4-tetrahydro-N-methyl-1-naphthalenamine hydrochloride, SRT, 342.69 g∙mol^−1^, 0.98 mass fraction purity) and randomly methylated β-cyclodextrin with Average Degree of Substitution DS ~12.5 (RMβCD) (~1310 g∙mol^−1^, 0.98 mass fraction purity) were purchased from Sigma-Aldrich (USA) or CycloLab (Hungary). The substances were used without any additional purifications. The solid substances were dried at 298 K for 72 h under reduced pressure. The water content in the cyclodextrin under investigation was determined as described previously [72]. Water used in the isothermal titration calorimetry and the circular dichroism spectroscopy measurements was distilled three times and degassed prior to experiments. 

### 3.2. Methods

#### 3.2.1. Isothermal Titration Calorimetry (ITC)

The isothermal titration calorimeter VP-ITC from MicroCal (Northampton, MA, USA) was used to carried out the calorimetric measurements in order to determine the thermodynamic parameters of interaction between sertraline hydrochloride and randomly methylated β-cyclodextrin molecules. The solubility of sertraline in water, even as hydrochloride salt is equal only to 3.8 mg mL^−1^ [36]. Such a low maximum possible concentration of SRT forces the drug solution to become a titrand placed in a measuring cell and RMβCD to get a titrant position in an injecting syringe [36,73]. Although cyclodextrin is a macromolecule it is used as a ligand and the drug plays a role of a macromolecule in the measuring cell, it is a very common situation and a more detailed justification for this is provided in the previous article [26]. Into a measuring cell (volume of 1.4275 mL) filled with an aqueous solution of SRT with concentration of 0.45 mM, a solution of RMβCD (15 mM) [74] was added by injection 55 portions of 5 µL titrant solution. Knowing from the literature, that methylated β-cyclodextrins could content from 4% to 6% *w/w* of water [61,62], the RMβCD has been dried under reduced pressure for 72 h in a Binder dryer till the moisture content were ≤1% *w*/*w* [72,75]. Prior to the ITC measurements, the solutions of the drug and the cyclodextrin were prepared separately by weighing with the use of a Mettler AE240 analytical balance [41] and degassed by ultrasounds during process of the real solutions preparation at 318 K and then cooled to the room temperature. 

The RMβCD concentration has been chosen as 15 mM inter alia in order to maintain similar measuring conditions as that for the ITC tests of SRT with the other non-ionic β-cyclodextrins [41,42]. Moreover, the concentration of RMβCD should not be greater than 15 mM for biological reasons (disruption of phospholipid membranes may be minimized if the concentration of RMβCD is kept below 15 mM [74]). Furthermore, when the concentration is higher there is a possibility of an aggregation process to occur [73,74,76]. In the presented case, the possible aggregation is slightly marked as a disaggregation during the cyclodextrin dilution (which can be seen in Figure 2 as the heat of RMβCD dilution equal to c.a. 5% of the whole enthalpic effect) and does not affect the course of the main interaction between the drug and the cyclodextrin. 

The titration of SRT solution by RMβCD solution was carried out with 380 s intervals between each injection, which took place within 10 s with a stirrer rotational speed of 264 rpm. The measurements were conducted at 298.15 K and pH 6.8. For getting the effects of direct interactions between SRT with RMβCD molecules in aqueous solution, the complementary to the main experiment measurements were carried out consisting of two stages:

1. the aqueous solution of the cyclodextrin was added into the pure water placed in the measurement cell and
2. the aqueous solution of sertraline hydrochloride was diluted with water injected from the syringe and the heat of the dilution for both stages were registered.

The subsidiary measurements were carried out with the use of the same procedure and the same concentration of the reagents as in the case of the main experiment. The obtained heats of dilutions were subtracted from the main titration data prior to the further proceedings and the example results of the main and the two subsidiary titrations are placed on Figure 2. After substruction, the proper value of the RMβCD:SRT interaction heat was analyzed as a function of the RMβCD/SRT ratio, and the data were fitted by a non-linear least squares method using the ORIGIN v.7.0 (USA) software [51] supplied with the calorimeter. Moreover, the first point (Figure 2) from first injection (3.0 μL) during the fitting sessions was discarded considering the circumstances described previously [26]. The calculated parameters were obtained as the average values from the five independent experiments, and the results were gathered in Table 1.

#### 3.2.2. Circular Dichroism (CD) Spectroscopy 

For circular dichroism spectroscopy measurements, the RMβCD and SRT were dried under reduced pressure for 72 h in a Binder dryer and dissolved in three times distilled and degassed water. The prepared aqueous mixtures of SRT and RMβCD by weighing with the use of a Mettler AE240 analytical balance [41] were placed in an ultrasonic washer until the solutions became clear and the stock solutions were stirred together for 15 min to obtain the mixtures with molar ratios from 1:0 to 1:33 of SRT:RMβCD for constant concentration of 0.3 mM for sertraline hydrochloride. A Jasco J-815 CD spectropolarimeter (Japan) has been used in order to measure the CD signals of the prepared solutions. The experiments were carried out at 298.15 K and the spectra were registered from 240 nm to 300 nm in 10-mm path length Helma quartz cuvettes. A wavelength step of 1 nm and a response time of 4 s have been chosen together with the scan rate of 50 nm/min. The final result was presented as an average calculated from three acquisitions. During the measurement the nitrogen was passed to cool and remove oxygen in order to avert ozone production inside the CD spectropolarimeter. Moreover, in order to compensate for baseline drift in CD spectra, a water blank sample was recorded.

#### 3.2.3. Computational Studies 

##### Ligands and Macromolecules Preparation for Molecular Docking

The X-ray structure with refcode: CAVVUQ01 [46] from Cambridge Structural Database (CSD) [47] were used in order to prepare the three-dimensional structure of SRT and protonated form of the drug was utilized for docking calculations. Likewise, crystal structures of used cyclodextrins for molecular modeling examinations were taken from the CSD with refcode: 648855 [67] for entries from Figure 7, Figure 8 and Figure 9, ALIGAE [68] for entries from Figure 10 and Figure 11 and JOSWOD [48] for entry from Figure 12. After removal of water and other ligands molecules, addition hydrogen atoms and Gasteiger charges to atoms [77,78] the docking procedure was carried out for cyclodextrin units [26].

##### Molecular Docking 

Docking examinations were carried out using Autodock Vina 1.1.2 software (The Molecular Graphic Laboratory, The Scripps Research Institute, La Jolla, CA, USA) [79]. For the docking studies the corresponding grid box parameters were used:

- entry from Figure 7 (three molecules of βCD I-II-III): a grid box size of 20 Å × 20 Å × 20 Å centered on the C47 atom (x = −5.017, y = 1.413, z = 0.074);
- entry A from Figure 8 (two molecules of βCD I-II): a grid box size of 20 Å × 20 Å × 20 Å centered on the C45 atom (x = 5.849, y = 3.007, z = −5.646);
- entry B from Figure 8 (two molecules of βCD II-III): a grid box size of 20 Å × 20 Å × 20 Å centered on the C23 atom (x = 4.807, y = 1.076, z = 7.878);
- entry A from Figure 9 (one molecule of βCD I): a grid box size of 20 Å × 20 Å × 20 Å centered on the C45 atom (x = 5.849, y = 3.007, z = −5.646);
- entry B from Figure 9 (one molecule of βCD II): a grid box size of 20 Å × 20 Å × 20 Å centered on the C43 atom (x = 5.243, y = 0.841, z = 1.262);
- entry C from Figure 9 (one molecule of βCD III): a grid box size of 20 Å × 20 Å × 20 Å centered on the C43 atom (x = 4.602, y = −1.221, z = 8.714);
- entry from Figure 10 (two molecules of TMβCD I-II): a grid box size of 20 Å × 20 Å × 20 Å centered on the C11 atom (x = 3.352, y = 6.710, z = 2.402);
- entry A from Figure 11 (two molecules of TMβCD I): a grid box size of 20 Å × 20 Å × 20 Å centered on the C11 atom (x = 3.352, y = 6.710, z = 2.402);
- entry B from Figure 11 (one molecule of TMβCD II): a grid box size of 20 Å × 20 Å × 20 Å centered on the C10 atom (x = 6.775, y = 12.684, z = 10.278);
- entry from Figure 12 (one molecule of RMβCD): a grid box size of 20 Å × 20 Å × 20 Å centered on the C45 atom (x = 2.967, y = 2.155, z = −4.366);

Graphic visualizations of the 3D model were generated using VMD 1.9 software (University of Illinois at Urbana—Champaign, Urbana, IL, USA).

## 4. Conclusions

The energetic effects of interaction between RMβCD and SRT molecules are exothermic—the enthalpy ΔH values are all less than zero and the enthalpic effects of binding have been dominated by the entropic effects (|ΔH| < |TΔS|) occurring during the drug-cyclodextrin complex formation. Moreover, the complexation process is spontaneous because the value of the Gibbs free energy of SRT binding with RMβCD is less than zero.

In the work presented, the enthalpy-entropy compensation effect has been observed for the non-ionic series of β-cyclodextrin derivatives interacted with sertraline hydrochloride molecules. From the results presented in the paper, one can conclude, that the different substituents in the studied β-cyclodextrins have an impact on the thermodynamical stability of the examined complexes. The intrinsic stability of the complex obtained will favored the complex formation even in the absence of the favorable enthalpy effects. The relationship between enthalpy and entropy factors confirms, that all considered SRT:βCDs complexes were not formed as a result of the covalent interactions. The binding constant for SRT included inside the RMβCD cavity is the lowest in comparison with the other non-ionic βCDs. Presumably, it is connected with the less enthalpy gain in the free Gibbs energy for that connection. The uneven distribution of methyl groups around the wider and narrower β-cyclodextrin edges has destabilizing effect during the guest-host complex formation. That could mean, the van der Waals interactions between SRT and RMβCD molecules are restricted by the less suitable fitting of SRT molecule inside the cyclodextrin cavity, since van der Waals forces are critically dependent on the distance of separation. The obtained complexation parameters of SRT interacting with βCD derivatives indicated that the thermodynamic parameters are sensitive functions of the amount, the position and type of the substituents introduced in the host molecule.

The significant difference in the magnitude of the free energies of binding for the 1:3 SRT:βCD complex obtained from molecular docking confirms that the connection of SRT:βCD gives the most stable complexes. Moreover, the free energies of binding for the 1:2 SRT:TMβCD complex are also more favorable than that obtained for 1:1 complex. Probably because of the possibility, which the cyclodextrin molecules possess to form the aggregates consisted of two and more molecules, in aqueous environment or the solid state. The possibility of the inclusion complexes formation between SRT and RMβCD with stoichiometry greater than 1:1 was also confirmed by the results obtained from the circular dichroism studies. Registered changes of CD signal, along with the increased concentration of RMβCD in relation to the constant SRT content, indicated the formation of the complexes with the stoichiometry 1:1.57 SRT:RMβCD, which reaffirms the stoichiometry coefficient estimated by ITC method (*n* = 1.26). The convergence of these data once more suggests that in the resulting complexes are more than one molecule of cyclodextrin per one drug molecule. 

The value of the constant K obtained by the CD method is slightly higher than that determined from the ITC experiments (K = 4520), and both methods showed that the formed complexes are stable at 298 K in aqueous solution.

To get a more complete picture of the interactions between sertraline hydrochloride and randomly methylated β-cyclodextrin, they may be investigated in the near future to see if the drug has better solubility in the presence of cyclodextrin or if it reduces the drug toxicity. Moreover, to make the conclusions about complexation thermodynamics more solid, the re-optimization of the free energy of the complexes with semi-empirical together with full density functional theory (DFT) optimization methods are in plans, as we did before for mianserin hydrochloride and β-cyclodextrin [25]. Knowing the constant interactions of SRT with various non-ionic β-cyclodextrins, it can be assumed to what extent and in what order the drug could be released from the complexes formed, which in the next step of the planned tests can be checked by means of the drug release examinations. 

## Figures and Tables

**Table 1:** Stoichiometry coefficients of binding (n), binding constants (K), enthalpic (ΔH), and entropic (TΔS) contributions into the Gibbs free energies values (ΔG) during the complex formation of sertraline hydrochloride molecules with some β-cyclodextrin derivatives at T = 298.15 K under atmospheric pressure *p* = 101,800 Pa obtained by ITC.

	*n*	*K*/M^−1^	ΔH/kJ·mol^−1^	TΔS/kJ·mol^−1^	ΔG/kJ·mol^−1^
β-CD ^a,b^	1.20 ^a^ 1.31 ^b^	5820 ^a^ 4999.3	−20.44 ^a^ −15.6 ^b^	1.06 ^a^ 5.5 ^b^	−21.53 ^a^ −21.1 ^b^
HPβCD ^c^	1.23	6530	−16.72	5.05	−21.77
DMβCD ^a^	1.60	7960	−14.20	7.96	−22.19
RMβCD *	1.26 ± 0.05	4520 ± 74	−8.37 ± 0.07	12.49 ± 0.04	−20.86 ± 0.11

^a^ Reference [41]. ^b^ Reference [36] with Corrigendum [36]. ^c^ Reference [42]. * This work. The uncertainties are standard deviation of an average value from five independent measurements.

**Table 2:** Stoichiometry coefficient of binding (*n*), binding constant (*K*) and maximum change in circular dichroism intensity (*ΔCD_max_*) together with reduced *χ*^2^ and *R*^2^ values for complex formation of sertraline hydrochloride with randomly methylated β-cyclodextrin molecules obtained from CD results at 274 nm.

*n*	*K*/M^−1^	*ΔCD_max_*/mdeg	Reduced *χ*^2^	*R* ^2^
1.57 ± 0.11	5315 ± 500	6.14 ± 0.05	0.00197	0.99976

reduced *χ*^2^—a deviation coefficient of the predicted values from the actual values in relation to the number of degrees of freedom—a small number of that coefficient indicates a good match of data and model, and *R*^2^—the coefficient of determination, the proportion of the variation in the dependent variable that is predictable from the independent variable—the value closer to 1 indicates that the regression predictions fit the data.

**Table 3:** The values of binding energies for SRT and chosen βCD molecules obtained from molecular docking results.

Representative Geometry	Crystal Structure Name (Refcode from CSD)	Free Energy of Binding kcal∙mol^−1^ (kJ∙mol^−1^)
Figure 7	648855	−9.2 (−38)
	(three molecules of βCD I-II-III)	
A	648855	−8.6 (−36)
Figure 8	(two molecules of βCD I-II)	
B	648855	−8.0 (−33)
Figure 8	(two molecules of βCD II-III)	
A	648855	−5.7 (−24)
Figure 9	(one molecule of βCD I)	
B	648855	−5.7 (−24)
Figure 9	(one molecule of βCD II)	
C	648855	−6.0 (−25)
Figure 9	(one molecule of βCD III)	
	ALIGAE	−7.4 (−31)
Figure 10	(two molecules of TMβCD I-II)	
A	ALIGAE	−6.5 (−27)
Figure 11	(one molecule of TMβCD I)	
B	ALIGAE	−5.8 (−24)
Figure 11	(one molecule of TMβCD II)	
	JOSWOD	−6.3 (−26)
Figure 12	(one molecule of RMβCD)	

## Data Availability

Not applicable.
